# Validation of the Comprehensive Geriatric Assessment as a Predictor of Mortality in Elderly Glioblastoma Patients

**DOI:** 10.3390/cancers11101509

**Published:** 2019-10-09

**Authors:** Giuseppe Lombardi, Eleonora Bergo, Mario Caccese, Marta Padovan, Luisa Bellu, Antonella Brunello, Vittorina Zagonel

**Affiliations:** 1 Department of Oncology, Oncology 1, Veneto Institute of Oncology IOV—IRCCS, 35128 Padua, Italy; giuseppe.lombardi@iov.veneto.it (G.L.);; 2 Radiation Therapy and Nuclear Medicine Unit, Veneto Institute of Oncology IOV—IRCCS, 35128 Padua, Italy

**Keywords:** elderly patients, glioblastoma, comprehensive geriatric assessment, temozolomide, radiotherapy

## Abstract

*Background:* Treatment of elderly glioblastoma patients (EGP) is a challenge in neuro-oncology. The comprehensive geriatric assessment (CGA) is currently used to assess geriatric oncological patients with other types of tumors. We performed a large retrospective study to analyze its predictive role in EGP. *Methods:* Patients aged ≥65 years with histologically confirmed diagnosis of glioblastoma were enrolled. CGA included the following tests: the Cumulative Illness Rating Scale-Comorbidity and Severity Index, Activities of Daily Living, Instrumental Activities of Daily Living, the Mini Mental State Examination, and the Geriatric Depression Scale. Based on CGA results, each patient was categorized as fit, vulnerable, or frail. *Results:* We enrolled 113 patients. According to the CGA scores, 35% of patients were categorized as “fit”, 30% as “vulnerable”, and 35% as “frail” patients. Median overall survival was 16.5, 12.1, and 10.3 months in fit, vulnerable, and frail patients (*p* = 0.1), respectively. On multivariate analysis, the CGA score resulted an independent predictor of survival; indeed, vulnerable and frail patients had a hazard ratio of 1.5 and 2.2, respectively, compared to fit patients (*p* = 0.04). No association between CGA and progression-free survival (PFS) was demonstrated. *Conclusions*: The CGA score proved to be a significant predictor of mortality in EGP, and it could be a useful treatment decision tool.

## 1. Introduction

Glioblastoma is the most common primary malignant brain tumor in adults. Approximately half of glioblastoma cases develop in geriatric patients, and this proportion will increase with the aging of the population [1,2]. Despite surgery, radiation therapy, and chemotherapy, glioblastoma prognosis still remains poor with a median overall survival of about 15 months for patients aged less than or equal to 70 years and about 9 months for elderly glioblastoma patients [3,4]. Indeed, in these patients, a more advanced age correlates with a worse prognosis [5]. However, other factors may contribute to the poor outcome in these patients: comorbid conditions, reduced treatment tolerance, treatment with less aggressive surgical and chemoradiation approaches, and tumor biology. Isocitrate dehydrogenase (IDH) mutations, predictive of favorable survival, are less common in the elderly compared to younger patients [6]. Historically, elderly cancer patients have been treated with standard or short-course radiotherapy or with temozolomide (TMZ) alone in the case of O6-methylguanine-DNA methyl-transferase (MGMT) gene promoter methylation, reporting a median overall survival of 5–9 months [7,8]. 

Recently, Perry et al. [4] demonstrated that the addition of temozolomide to short-course radiotherapy in patients with glioblastoma aged 65 years and older provided significantly longer survival compared to short-course radiotherapy alone. This benefit was greater in patients with methylated versus unmethylated MGMT.

However, management of elderly glioblastoma patients (EGP) is challenging due to the disease-specific poor prognosis, impaired neurological status, comorbid conditions, and higher risk of treatment-related adverse events; together, these aspects cause concern over the balance between treatment benefits, treatment-related side effects, and quality of life. Therefore, the question whether all elderly patients can receive combined chemoradiation therapy still remains open. 

A careful evaluation of the global health status of elderly patients could be helpful in identifying patients who could benefit from specific treatments. The use of a comprehensive geriatric assessment (CGA), which involves evaluation of the functional, nutritional, cognitive, and psycho-social status of elderly patients, has indeed been recommended by the International Society of Geriatric Oncology (SIOG) for elderly cancer patients. Systematic application of CGA could help in tailoring therapy, avoiding over- and under-treatment.

The CGA, introduced in oncology around the year 2000 [9,10], was subsequently applied in several series [11,12] and has shown both prognostic [13] and predictive ability in the general population of elderly cancer patients [14]. Classical CGA, according to Balducci’s criteria, classifies patients in good general health condition as “fit”, patients with partial impairment in some domains as “vulnerable”, and patients with severe impairment in most domains or dependency in activities of daily living as “frail”. Such a classification has been demonstrated to hold a prognostic significance [15,16].

To the best of our knowledge, no study has analyzed CGA as predictor of mortality in EGP. We therefore performed a retrospective study to assess the prognostic ability of CGA in elderly glioblastoma patients. 

## 2. Material and Methods

This is a retrospective study on patients with newly diagnosed GBM, consecutively treated at the Veneto Institute of Oncology, IRCCS, Medical Oncology 1, from January 2011 to December 2017. Inclusion criteria were: patients aged ≥65 years and histologically confirmed diagnosis of GBM; patients with a secondary glioblastoma were excluded. Data were extracted by a prospectively maintained database. 

CGA, as per institutional policy, was administered to all new GBM patients aged 65 years and older, generally 3–4 weeks after surgery. The evaluation was carried out by a trained psychologist through a clinical interview with the patient and family members. 

The ultimate decision for treatment was made based on the oncologist’s expertise, given the lack of CGA-based guidelines for treatment of patients with GBM. In particular, for patients with a Karnofsky Performance Status (KPS) between 60 and 40, TMZ monotherapy was recommended in case of methylated MGMT, while RT alone in presence of unmethylated MGMT, according to NOA-08 [8] and Nordic [17] trial results; indeed, in both trials, temozolomide was associated with survival advantages compared to radiotherapy in elderly patients with MGMT promoter methylation. 

Baseline gadolinium-enhanced brain MRI was performed before starting treatment, and subsequent assessments were performed every 2–3 months or when clinically indicated. To exclude pseudoprogression in brain-MRI images performed within 12 weeks after completing the RT and TMZ treatment, disease progression was defined as appearance of new enhancement outside the radiation field. All MRI evaluations were performed according to RANO criteria

The CGA included the following tests: the Cumulative Illness Rating Scale-Comorbidity Index (CIRS-CI) and Severity Index (CIRS-SI) [18], Activities of Daily Living (ADL) [19], Instrumental Activities of Daily Living (IADL) [20], the Mini Mental State Examination (MMSE) [21], and the Geriatric Depression Scale (GDS) [22]. Geriatric syndromes were also considered. 

Based on CGA results, according to Balducci’s criteria, [23] patients were considered “fit” if functionally independent and without serious comorbidity; “vulnerable” if dependent for one or more IADLs and/or presenting with one or two non-severe comorbidities; and “frail” if presenting with 3 or more grade 3 comorbidities or at least 1 grade 4 comorbidity, and/or with some geriatric syndrome and/or dependent for at least 1 ADL item, and/or if aged ≥85 years.

Besides CGA items, data were also collected on patient characteristics; Karnofsky Performance Status (KPS); tumor molecular status; date and type of surgery (biopsy, partial, radical surgery); treatment received (radiotherapy, chemotherapy, radio-/chemotherapy, cycles of maintenance therapy, or the best supportive care); date of progression or last follow up; and date of death or last follow up.

This study was approved by the local ethics committee and was conducted in accordance with the ethical standards as per the Declaration of Helsinki.

## 3. Statistical Analysis

We analyzed prognostic factors and clinical outcomes in terms of median progression-free survival (PFS) and median overall survival (OS). PFS was calculated from diagnosis until disease progression or death from any cause, or the last day of follow up. OS was calculated from diagnosis to the date of death from any cause or to the last day of follow up. 

PFS and OS were described using Kaplan–Meier survival curves. In addition to CGA categories, PFS and OS were stratified according to well-known prognostic factors in glioblastoma patients: treatment with RT and TMZ (yes versus no), radical surgery (yes versus no), MGMT methylation status (methylated versus unmethylated), and Karnofsky Performance Status (≥70 versus ≤60). 

A multivariate Cox proportional hazards regression model was used for univariate and multivariate analysis to test the effect of prognostic factors in terms of overall survival and progression-free survival. For the multivariate models, a univariate inclusion criterion of *p* ≤ 0.2 was used.

Fisher exact test was used to evaluate the correlation between some clinical characteristics and CGA categories.

*p* values were based on two-side testing, and differences with a *p* ≤ 0.05 were considered significant. All statistical analyses were performed using SPSS version 22 statistical software (SPSS Inc., Chicago, IL, USA).

## 4. Results

We enrolled 113 patients. Baseline patient characteristics are shown in Table 1.

The median age was 71.7 years (range 65–84), and male patients represented the majority of our cohort (64%). Radical surgery was performed in 33% of patients; 80% of patients were treated with a radio-/chemotherapy combination (40% and 60% of patients received hypofractionated and standard radiation therapy, respectively) while single therapy (radiotherapy or temozolomide alone) was administered in 14% of cases; the median number of maintenance TMZ cycles was 3.9; best supportive care was performed in 6% of the patients. Most patients had a high KPS (80%) and unmethyalted status of MGMT (56%).

According to CGA, 35% of patients were classified as “fit”, 30% as “vulnerable”, and 35% as “frail”. KPS scores correlated with patient status according to CGA categories (*p* < 0.001) (Table 2). 

CGA-based classification was significantly associated with treatment type: a combination treatment was used for 98% of fit patients, 90% of vulnerable patients, and 52% of frail patients (*p* < 0.001); (Table 2). It is noteworthy that frail patients received fewer cycles of maintenance temozolomide compared to vulnerable and fit patients: 2.8 vs. 5 vs. 5.2 (*p* = 0.03), respectively. All fit patients (100%) received temozolomide (94% and 85% of vulnerable and frail patients, *p* = 0.03). Although more fit patients (45%) than frail patients (22%) were treated with radical surgery, and biopsy was performed more frequently among frail patients (7%) compared to fit (0%) or vulnerable patients (3%), the type of surgery did not show a statistically significant correlation with CGA categories (Table 2). Among cases analyzed for MGMT methylation status (96 cases), 44% of fit patients and 50% of vulnerable and frail patients had methylated MGMT.

**Figure 1:**
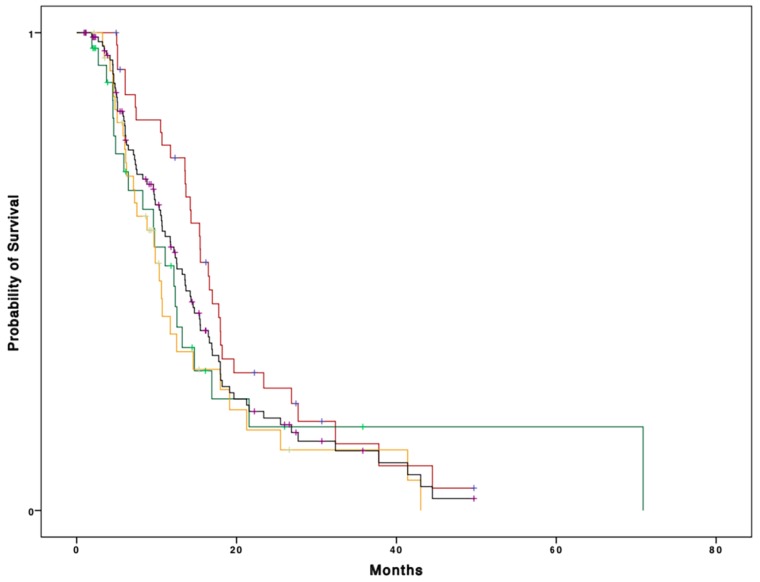
Kaplan–Meier curve representing overall survival (OS) according to CGA categories (fit patients: red line; vulnerable patients: green line; frail patients: orange line; whole population: black line).

With a median follow up of 30.6 months, median OS in the whole cohort of patients was 13.2 months (95% CI 10.9–15.4) (Figure 1). Median OS for fit patients was 16.5 months (95% CI 14.6–18.2), whereas it was 12.1 months (95% CI 8.1–16.1) and 10.3 months (95% CI 8.8–11.8) for vulnerable and frail patients, respectively. This difference was not significant on univariate analysis. (*p* = 0.1) (Figure 1).

Median OS for all patients was 13.2 months (95% CI 10.9–15.4). Median OS was 16.5 months (95% CI 14.6–18.2), 12.1 months (95% CI 8.1–16.1), and 10.3 months (95% CI 8.8–11.8) for fit, vulnerable, and frail patients (*p* = 0.1), respectively. 

Yet, when grouping vulnerable and frail patients into an “unfit” group, a statistically significant difference in survival was observed, with median OS for fit patients of 16.5 months (95% CI 14.6–18.2) compared to 10.6 months (95% CI 8.3–12.8) for unfit patients, *p* = 0.04.

Other factors significantly associated with better survival were the presence of methylated MGMT, good KPS, and treatment with chemoradiotherapy (Table 3).

On multivariate analysis, adjusted for type of surgery, MGMT methylation status, KPS, and type of therapy, CGA score was significantly associated with survival, with vulnerable and frail patients having worse survival (HR 1.5 (95% CI 1.1–2.09; *p* = 0.05) and HR 2.2 (95% CI 1.2–5.4), respectively) compared to fit patients (*p* = 0.04) (Table 4). MGMT methylation status and KPS remained as independent predictors for survival. 

Furthermore, when considering “unfit” patients, the association with worse survival was confirmed compared to fit patients (HR 1.8; 95% CI 1.1–2.8).

**Figure 2:**
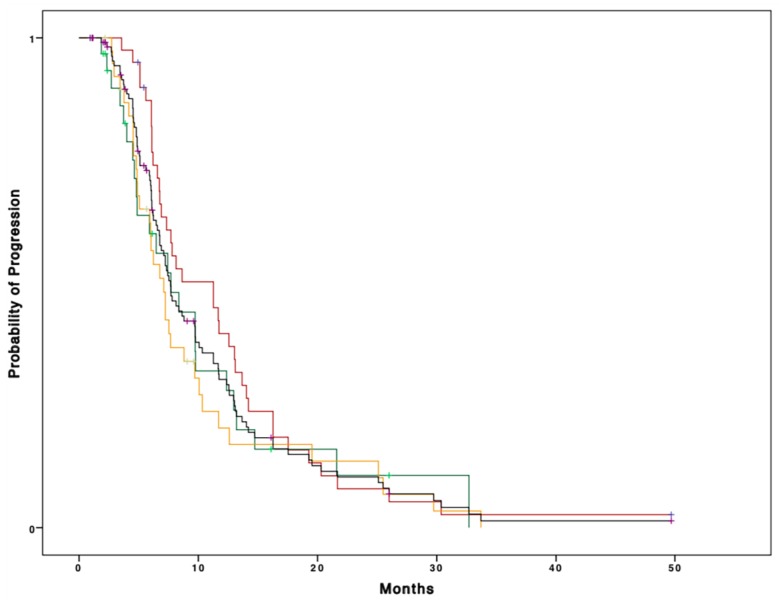
Kaplan–Meier curve representing the PFS according to CGA categories (fit patients: red line; vulnerable patients: green line; frail patients: orange line; whole population: black line).

As for progression-free survival, the median PFS in the whole cohort of patients was 7.6 months (95% CI 6.5–8.7). On univariate analysis, no significant difference (*p* = 0.2) was shown between CGA categories, with fit patients having a median PFS of 11.2 months (95% CI 6.07–16.4), vulnerable patients of 7.7 months (95% CI 4.6–10.7), and frail patients of 7.1 months (95% CI 5.7–8.4) (Figure 2). 

Median PFS for all patients was 7.6 months (95% CI 6.5–8.7). Median PFS was 11.2 months (95% CI 6.07–16.4), 7.7 months (95% CI 4.6–10.7), and 7.1 months (95% CI 5.7–8.4) for fit, vulnerable, and frail patients (*p* = 0.2), respectively.

No difference was observed even after grouping vulnerable and frail patients into the “unfit” category on univariate analysis (Table 3). No difference according to CGA categories was observed on multivariate analysis either (Table 4).

As for the other potential prognosticators, methylated MGMT status and treatment with chemoradiation were shown to be independent predictors of longer PFS (Table 4).

## 5. Discussion

To the best of our knowledge, the present study is the first report considering the role of the CGA as a prognosticator in elderly patients with glioblastoma.

After Perry et al. [4] demonstrated the benefit from a combination treatment with radiotherapy and temozolomide followed by 12 cycles of maintenance temozolomide in glioblastoma patients aged ≥65 years, especially those with methylated MGMT, this treatment became the standard therapy for EGP. However, real-world practice involves dealing with potentially less fit patients compared to those enrolled in clinical trials, and decision-making is often difficult due to several factors, such as the presence of neurological disorders, comorbidities, performance status, and patient adherence. Age alone should not be a criterion for decision-making, yet in some cases an aggressive approach with combination treatment could be detrimental to specific patients, leading to decreased survival and quality of life.

CGA has been long used by geriatricians to properly evaluate elderly patients, to prevent worsening of disability and recurrent falls, and to reduce mortality. In the past 20 years, several studies have also applied a CGA-based approach to oncology, demonstrating a prognostic and predictive role in unselected elderly cancer patients [13,24,25]. The International Society of Geriatric Oncology (SIOG) has advocated the use of CGA in cancer patients [26]. Likewise, the American Society of Clinical Oncology recently published its guidelines on the assessment and management of vulnerable elderly patients, and the panel of experts recommended that “in patients ≥ 65 years who receive chemotherapy, geriatric evaluation should be used to identify vulnerabilities that are not detected daily during the oncological assessment” and “the geriatric assessment should be applied to develop and integrate an individualized patient care plan” [27].

Our data show that CGA-based categories were statistically and independently correlated with overall survival, with frail and vulnerable patients having a greater probability of shorter survival compared to fit patients. Taking into account these data, patients classified as “fit” are those who should be offered a chemoradiation approach given their longer overall survival.

Our results showed a prevalence of frailty in EGP of 35%, which is much higher than what is expected in the overall elderly population; according to a previous systematic review of frailty in community-dwelling older adults, data collected using CGA demonstrated 13.6% as frail patients [28]. On the other hand, comparing our results to elderly patients with other tumor types, we can see a lower prevalence of frailty and a higher rate of fit patients among EGP; a recent study categorized 745 patients with gastrointestinal, breast, and urological cancer as “frail” in 45%, as “vulnerable” in 34%, and as “fit” in 20% of the cases [29]

Although the choice of treatment was not based on the results of the CGA, the three CGA categories were statistically associated with the use of a radio-/chemotherapy combination; moreover, there was also an association with the Karnofsky Performance Status and the number of maintenance temozolomide cycles. Interestingly, frail patients received only 2.8 cycles of temozolomide after chemoradiotherapy due to progressive disease, toxicity, or death. Although KPS is a well-established prognostic factor for glioblastoma patients and we showed an association between KPS and CGA categories, CGA is a useful instrument which adds information to the performance status in the decision making for elderly cancer patients [9,30,31]. In our series, 47% of patients who were deemed frail at CGA had a good KPS between 70 and 100. Based on KPS, these patients were indeed treated with combination radiotherapy, given the lack of CGA-based guidelines for treatment in GBM elderly patients. Results of this study suggest, however, that these patients may have been overtreated. EGPs have considerable variability in outcome that cannot be accounted for by KPS alone, but multiple aspects of physical health need to be investigated. Our CGA, involving a multidimensional assessment of the medical, psychological, and functional capability of EGP, adds information to oncological standard functional assessment, such as performance status. Moreover, physical function is a core measure upon which physicians decide about oncologic treatment and it is part of our CGA by ADL and IADL tests. In addition, our CGA was associated with an outcome independent of KPS, type of treatment, and MGMT methylation status.

Conversely, CGA was not a predictor of PFS; this result is in line with another study analyzing the comorbidity assessment in elderly patients with glioblastoma treated with radiochemotherapy [32]. The lack of association between CGA categories and PFS could be biased by the globally low PFS rates. In the study by Fiorentino and colleagues [32], the Adjusted-Age Charlson Comorbidity Index was used to assess comorbidity in 35 elderly patients with glioblastoma, and those with a low score experienced a longer survival time than those with a higher score (22 and 10 months, respectively); yet, no difference in PFS was observed between the two categories. However, the study by Fiorentino et al. was a small retrospective study reporting two categories of patients (low and high comorbidity).

Our study, compared to that of Fiorentino and colleagues, was based on a more representative patient population, given the higher sample size, and took into account all the potentially impaired domains in elderly patients, not just comorbidity, allowing for a more precise patient classification. Recently, Deluche et al. [30] demonstrated the role of the screening tool G8 in predicting prognosis in elderly patients with newly diagnosed glioblastoma; they reported a median OS of 8.0 months in patients with abnormal G8 score and 42 months in those with a normal G8 score (*p* < 0.0001). Although patients with impaired G8 had a HR of 10.27, the difference in overall survival was too wide between the two groups of patients (∆ = 34 months); therefore for a better identification of patients with a poor prognosis, the authors classified the patients into 3 categories (low, intermediate, and high score group), demonstrating a statistically different OS among these groups; however, contrary to expectations, “intermediate” patients reported a higher HR compared to those of the high score group (55.46 versus 8.6, respectively). In our study, the HR of “frail” was higher than “vulnerable” patients, as expected. Unlike the work by Deluche et al. [30], we also analyzed the role of MGMT, an important prognostic and predictor factor in EGP; our univariate and multivariate analyses included MGMT methylation status.

Globally, our study mostly included patients with good KPS (80%) and this may explain the longer PFS and OS that we observed in our series compared to data on elderly glioblastoma patients from phase III randomized studies [4,8,17].

CGA might also be used for surgical risk assessment in elderly glioblastoma patients to avoid an aggressive surgical approach in the case of frail patients. This should be focused upon in a prospective manner. Indeed, a prior retrospective study examined frailty on surgical outcomes in geriatric glioblastoma patients with the Canadian Study of Health and Aging Modified Frailty Index; the authors concluded that frailer patients were less likely to undergo surgical resection compared to biopsy, reported longer hospital stays, major complications, and shorter overall survival [31].

Furthermore, although various studies confirmed the optimal role of hypofractionated compared to standard radiotherapy in EGP [4,7,17], there are some concerns on whether patients aged 65–70 years can benefit from standard treatment; in this case, CGA could help physicians choose the most suitable treatment, using standard radiotherapy for fit patients only and thus avoiding overtreatment and toxicity in frail or vulnerable patients.

In addition to CGA, integration of clinical outcome assessments such as health-related quality of life (HRQOL) metrics and patient-reported outcomes (PROs) could provide reliable data on quality of life and impact of treatment on patient benefit. PRO measures can assess multiple domains of HRQOL, symptoms and functional limitations known only to the patient, via self-report. The benefit of a treatment in terms of prolonged survival has to be weighed against the side-effects of therapy; in a prior study, it was demonstrated that monitoring symptoms via PROs can be very helpful for patients and even influence survival [33]. Therefore, the use of PROs in EGP during therapy could improve treatment management, symptom control, and quality of life. Choosing the optimal types of PRO measures for identifying most signs, symptoms, and functional limitations identified as priorities for EGP remains a challenging issue. In fact, due to neurocognitive deficits and poor clinical conditions of EGP, noncompliance with PRO assessment is frequently reported, which could make these tools not easily applicable in this setting of patients [34].

Our study has some limitations. It was a retrospective study and we excluded patients without histological diagnosis of GBM. Likewise, patients with a very poor clinical condition were excluded due to the inability to undergo surgery or biopsy.

## 6. Conclusions

In conclusion, based on our data, CGA held prognostic significance in elderly patients with glioblastoma. It is likely that CGA fit patients are those who would benefit from combined treatment with radiochemotherapy. To confirm this hypothesis, a prospective study is warranted to prospectively assess both the role of chemoradiation in fit patients and of single treatment (either radiotherapy or temozolomide alone) versus supportive care in vulnerable or frail patients. Prospective studies should be done to determine whether using CGA categories to dictate treatment has an effect on survival and quality of life. Moreover, MGMT promoter methylation status being an important predictor to temozolomide benefit, vulnerable patients could be treated with radiotherapy or temozolomide alone based on their MGMT methylation status. 

## Figures and Tables

**Table 1:** Baseline characteristics of patients.

Variable	Category	N (%)
Number of patients	113	
Age at diagnosis	Mean ± SD	71.7 ± 4.6
	Median	71.3 (range 65–84)
Gender	Male	72 (64)
	Female	41 (36)
MGMT	Methylated	42 (44)
	Not methylated	54 (56)
KPS	100–70	90 (80)
	60–30	23 (20)
Type of surgery	Radical	37 (33)
	Partial	72 (63)
	Biopsy	4 (4)
Treatment	Yes	106 (94)
	No	7 (6)
Type of treatment	RT + TMZ	90 (80)
	RT 60 Gy (standard)	54 (60)
	RT 40 Gy (hypofractionated)	36 (40)
	TMZ or RT alone	16 (14)
	Best supportive care	7 (6)
	Maintenance TMZ cycles (median)	3.9
CGA	Fit	40 (35)
	Vulnerable	33 (30)
	Frail	40 (35)

MGMT = O6-methylguanine-DNA methyl-transferase; RT = radiotherapy; TMZ = temozolomide; CGA = multidimensional geriatric assessment; KPS: Karnofsky Performance Status.

**Table 2:** Association of clinical factors and CGA categories.

Variables	Fit	Vulnerable	Frail	*p*
RT+TMZ	39/40 (98%)	30/33 (90%)	21/40 (52%)	**<0.001**
RT 60 Gy (standard)	25/29 (64%)	21/30 (70%)	8/21 (38%)	0.06
RT 40 Gy (hypofractionated)	14/39 (36%)	9/30 (30%)	13/21 (62%)	0.06
KPS 100–70	40/40 (100%)	31/33 (94%)	19/40 (47%)	**<0.001**
Maintenance TMZ Cycles (median)	5.2	5	2.8	**0.03**
Administration of TMZ	40/40 (100%)	31/33 (94%)	34/40 (85%)	**0.03**
Radical Surgery	18/40 (45%)	10/33 (33%)	9/40 (22%)	0.09
Biopsy	0/40 (0%)	1/33 (3%)	3/40 (7%)	0.2
Methylated MGMT	15/34 (44%)	16/32 (50%)	15/30 (50%)	0.8

RT = radiotherapy; TMZ = temozolomide; KPS = Karnofsky Performance Status; Gy = Gray; MGMT = O6-methylguanine-DNA methyl-transferase. In bold are shown statistically significant values (≤0.05).

**Table 3:** Univariate analyses of clinical factors associated with progression-free survival (PFS) and OS.

Univariate Analysis							
Variables		PFS			OS		
		Median (ms)	95% CI	*p*	Median (ms)	95% CI	*p*
**CGA**				0.2			0.1
	Fit	11.2	6.07–16.4		16.5	14.6–18.2	
	Vulnerable	7.7	4.6–10.7		12.1	8.1–16.1	
	Frail	7.1	5.7–8.4		10.3	8.8–11.8	
**CGA** **(fit vs. unfit)**				0.25			**0.04**
	Fit	11.2	6.07–16.4		16.5	14.6–18.2	
	Unfit	7.2	5.8–8.6		10.6	8.3–12.9	
**MGMT**				**0.002**			**0.01**
	met	11.7	8.8–14.5		16.4	11.9–20.9	
	unmet	7.2	6.3–8.1		12.1	9.7–14.5	
**Radical surgery**				0.3			0.1
	yes	10.3	7.3–13.3		14.73	11.9–17.5	
	no	7.1	6.2–7.9		10.7	7.9–13.5	
**KPS**				**<0.001**			**0.008**
	100–70	9.4	6.8–11.9		14.3	12.05–16.5	
	≤60	6.0	4.9–7.04		10.3	3.9–16.6	
**RT+TMZ**				**0.006**			**0.001**
	yes	8.1	6.1–10.1		14.3	12.4–16.1	
	no	6	2.5–9.4		8.2	5.7–10.8	

MGMT = O6-methylguanine-DNA methyl-transferase; RT = radiotherapy; TMZ = temozolomide; CGA = multidimensional geriatric assessment; KPS: Karnofsky Performance Status: met = methylated; unmet = unmethylated. In bold are shown statistically significant values (≤0.05).

**Table 4:** Multivariate analyses of clinical factors associated with PFS and OS.

Multivariate Analysis						
Variables	PFS			OS		
	HR	95% CI	*p*	HR	95% CI	*p*
**CGA**						
Fit	Rif.			Rif.		
Vulnerable	1.1	0.4–1.7	0.7	1.5	1.1–2.09	**0.05**
Frail	1.6	0.7–3.3	0.2	2.2	1.2–5.4	**0.04**
**CGA** (unfit vs. fit)	-	-	-	1.8	1.2–2.8	**0.02**
**MGMT** (met vs. unmet)	0.4	0.2–0.8	**0.009**	0.4	0.2–0.7	**0.001**
**Radical Surgery** (yes vs. no)	-	-	-	0.9	0.7–1.2	0.7
**KPS** (100–70 vs. ≤60)	0.4	0.1–0.8	**0.01**	0.4	0.2–0.9	**0.05**
**RT+TMZ** (yes vs. not)	0.7	0.3–1.5	0.4	0.8	0.4–1.5	0.5

MGMT = O6-methylguanine-DNA methyl-transferase; RT = radiotherapy; TMZ = temozolomide; CGA = multidimensional geriatric assessment; KPS = Karnofsky Performance Status; met = methylated; unmet = unmethylated. In bold are shown statistically significant values (≤0.05).
